# *dachshund* Potentiates Hedgehog Signaling during *Drosophila* Retinogenesis

**DOI:** 10.1371/journal.pgen.1006204

**Published:** 2016-07-21

**Authors:** Catarina Brás-Pereira, Delphine Potier, Jelle Jacobs, Stein Aerts, Fernando Casares, Florence Janody

**Affiliations:** 1 Instituto Gulbenkian de Ciência, Oeiras, Portugal; 2 Department of Human Genetics, KULeuven, Leuven, Belgium; 3 Centro Andaluz de Biología del Desarrollo (CABD), CSIC-UPO, Seville, Spain; New York University, UNITED STATES

## Abstract

Proper organ patterning depends on a tight coordination between cell proliferation and differentiation. The patterning of *Drosophila* retina occurs both very fast and with high precision. This process is driven by the dynamic changes in signaling activity of the conserved Hedgehog (Hh) pathway, which coordinates cell fate determination, cell cycle and tissue morphogenesis. Here we show that during *Drosophila* retinogenesis, the retinal determination gene *dachshund (dac)* is not only a target of the Hh signaling pathway, but is also a modulator of its activity. Using developmental genetics techniques, we demonstrate that *dac* enhances Hh signaling by promoting the accumulation of the Gli transcription factor Cubitus interruptus (Ci) parallel to or downstream of *fused*. In the absence of *dac*, all Hh-mediated events associated to the morphogenetic furrow are delayed. One of the consequences is that, posterior to the furrow, *dac-* cells cannot activate a Roadkill-Cullin3 negative feedback loop that attenuates Hh signaling and which is necessary for retinal cells to continue normal differentiation. Therefore, *dac* is part of an essential positive feedback loop in the Hh pathway, guaranteeing the speed and the accuracy of *Drosophila* retinogenesis.

## Author Summary

Molecules of the Hedgehog (Hh) family are involved in the control of many developmental processes in both vertebrates and invertebrates. One of these processes is the formation of the retina in the fruitfly *Drosophila*. Here, Hh orchestrates a differentiation wave that allows the fast and precise differentiation of the fly retina, by controlling cell cycle, fate and morphogenesis. In this work we identify the gene *dachshund* (*dac*) as necessary to potentiate Hh signaling. In its absence, all Hh-dependent processes are delayed and retinal differentiation is severely impaired. Using genetic analysis, we find that *dac*, a nuclear factor that can bind DNA, is required for the stabilization of the nuclear transducer of the Hh signal, the Gli transcription factor Ci. *dac* expression is activated by Hh signaling and therefore is a key element in a positive feedback loop within the Hh signaling pathway that ensures a fast and robust differentiation of the retina. The vertebrate *dac* homologues, the DACH1 and 2 genes, are also important developmental regulators and cancer genes and a potential link between DACH genes and the Hh pathway in vertebrates awaits investigation.

## Introduction

Temporal and spatial coordination between cell proliferation and differentiation is essential for proper organ patterning. A way to ensure this coordination is through the use of regulatory signaling pathways that control both processes. Among those is the Hedgehog (Hh) signaling pathway that regulates organ growth and patterning in embryos and tissue homeostasis in adults, both in vertebrates and invertebrates [1–4]. Not surprisingly, mutations in components of the Hh signaling pathway cause a number of human disorders, including congenital abnormalities and cancer [2–4].

**Fig 1:**
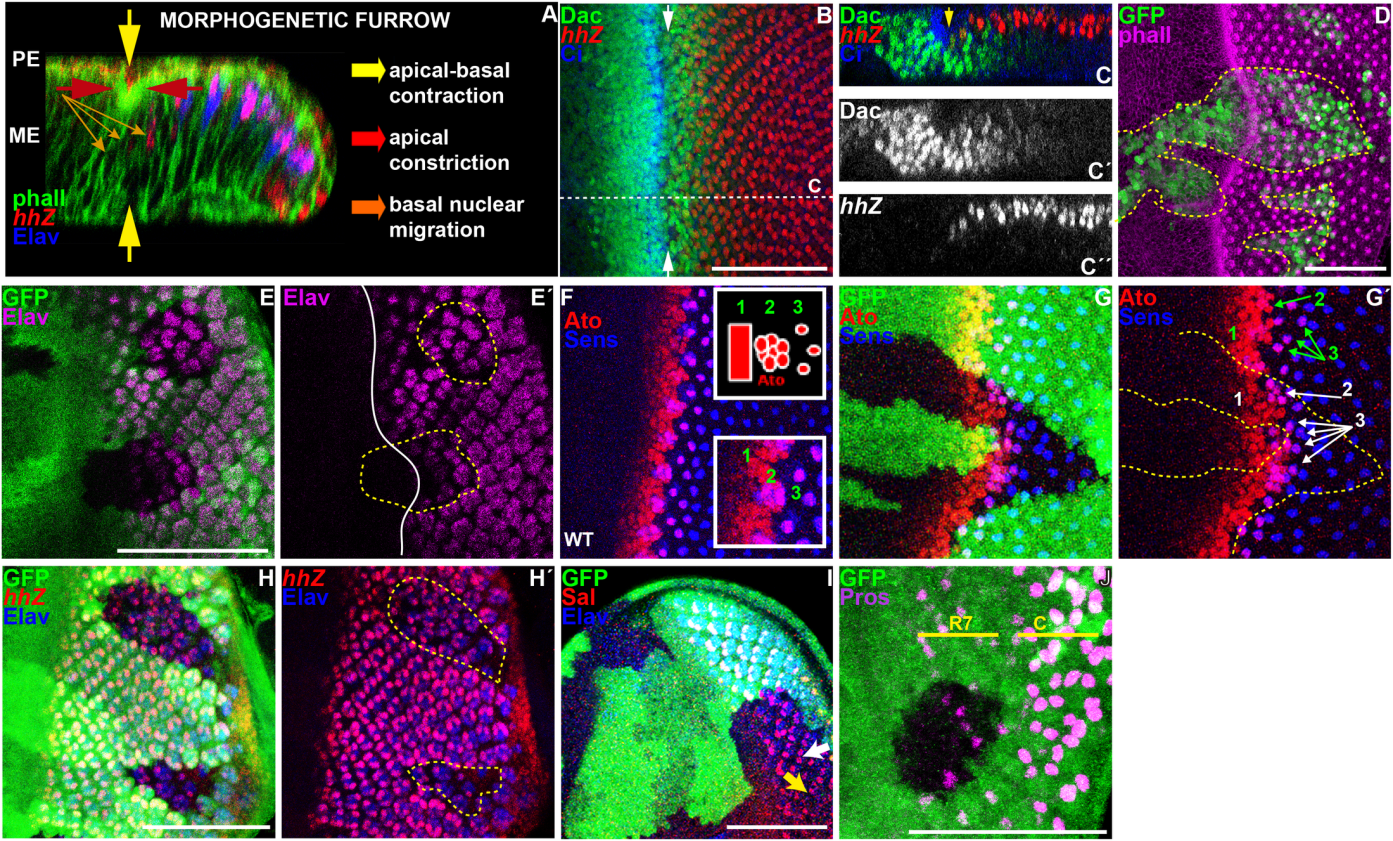
Loss of *dac* function affects the dynamics of the MF and proper retinogenesis. All panels show L3 eye imaginal discs. (A) Cross-section through a wild type disc with anterior to the left and apical side up, revealing that the formation of the MF depends on the coordination of three different cell processes: apical constriction (red arrow), apical-basal contraction (yellow arrow), seen by phalloidin staining, which outlines cell shape (green) and nuclear basal migration (orange arrow), labeled with anti-Elav (blue), which marks PR nuclei and anti-β-Galactosidase to detect *hhZ* (red). (B,D-J) Standard confocal sections with anterior to the left and dorsal up on this and all subsequent eye disc panels. (C-C´´) Cross section through the wild type disc in B (dashed white line). (B,C-C´´,F) wild type discs stained with (B,C-C´´) anti-Dac (green in B,C and white in C´), anti-β-Galactosidase to detect *hhZ* (red in B,C and white in C´´) and anti-Ci (blue in B,C) or (F) anti-Ato (red) and anti-Sens (blue). Note that Dac is expressed at high levels in a stripe that comprises the MF (arrows in B,C). (F) Ato shows three distinct expression pattern: ahead of the MF, all cells express Ato (1), posterior to the MF, Ato is restricted to a group of cells that start to co-express Sens (2). More posterior, its expression is only maintained in R8 cell until the 2^nd^ row of cells (3), while Sens expression persists onwards [21,62,88,93,94]. (D-E´ and G-J) *dac*^*3*^ clones marked by the (D) presence or (E-E´ and ´G-J) absence of GFP (green) and outlined by yellow dashed lines in D,E´,G´ and H´. Discs are stained with (D) phalloidin (magenta) or (E-E´) anti-Elav (magenta) or (G-G´) anti-Ato (red) and anti-Sens (blue) or (H-H´) anti-Elav (blue) and anti-β-Galactosidase (red) to reveal *hhZ* or (I) anti-Elav (blue) and anti-Sal (red) or (J) anti-Pros (magenta), R7 indicates PR7 and C indicates cone cells. Plain white line in E’ marks the MF. Note that in *dac* mutant clones, the refinement of Ato expression is atypical: its expression is not restricted to the groups of cells that start to co-express Sens (2) and is not singled out properly in R8 cells (3) (1G´, compare green with white annotations). *dac* mutant ommatidia show irregular number of cells: with only one (white arrow in I) or 2 Sal-expressing PR (yellow arrow in I) and fewer PRs (Elav positive cells, E-E´, H-H´). Scale bars represent 50μm.

One of the processes in which Hh signaling plays an essential role is the patterning of the retina in vertebrates and invertebrates [5–7]. In *Drosophila*, Hh is responsible for organizing a moving signaling wave that patterns the primordium of the fly eye during the last larval stage (L3). The processes under Hh signaling control have been extensively studied and summarized in what follows. The front of the differentiation wave is marked by a straight indentation of the eye epithelium, called morphogenetic furrow (MF), that runs across the dorsoventral axis of the eye primordium, or “eye imaginal disc” [8–10]. *hh*, initially expressed along the posterior margin of the eye disc [11] and later by the differentiating photoreceptors (PRs), activates the expression of the BMP2 *decapentaplegic (dpp)* within the MF [12]. Hh instructs undifferentiated proliferating progenitor cells to synchronously undergo mitosis (First Mitotic Wave, FMW) and then stop temporarily their cell cycle in G1 phase through Dpp, which acts long range [13–16]. At a shorter range, Hh initiates the expression of the proneural gene *atonal (ato)* [17–23] and stabilizes the G1 state by activating the expression of the p21/p27 Cdk inhibitor homologue *dacapo (dap)* [24–27]. In addition, together with Dpp, Hh induces coordinated cell shape changes responsible for MF formation [18,19,23,28–31] by promoting the apical constriction, apical-basal contraction and basal nuclei migration of cells (Fig 1A). These cellular changes are mediated, at least in part, through the contraction of the acto-myosin cytoskeleton [32,33]. Immediately behind the MF, Ato expression is restricted to evenly spaced cells, which become the ommatidial founder photoreceptors (PR8s). Then, PR8s induce neuronal differentiation of the adjacent precursor cells. Precursor cells that did not start their differentiation program immediately after the MF suffer one last round of mitosis, the Second Mitotic Wave (SMW) [10]. Therefore, Hh secreted by differentiating PR cells drives the anterior propagation of the MF and its associated differentiation wave, while regulating the SMW locally [25,34–36]. Thus, the MF coincides spatially with the onset of differentiation. Interestingly, the MF state is transient: while anterior precursor cells are recruited to enter the MF state, the newly differentiating PRs and cells at the SMW exit this “furrowed” state.

The coordinated action of Hh has been shown to rely on dynamic changes of its signaling activity. In flies, Hh signaling regulates the post-translational proteolytic processing of the Gli-family transcription factor Cubitus interruptus (Ci). Hh binding to the receptor Patched (Ptc) relieves the inhibition exerted by unbound Ptc on the transducer Smoothened (Smo), and thus promotes the activation of the Fused (Fu) kinase [1–3]. In turn, activated Fu promotes the conversion of the full-length form of Ci (Ci^FL^) to Ci activator (Ci^A^) form [37]. As a result, Ci^FL^ is no longer phosphorylated by Protein Kinase A (PKA) [38–40] and other kinases. Otherwise, Ci^FL^ phosphorylation leads to the generation of the Ci repressor form (Ci^R^) through partial Ci^FL^ degradation by the F-box-containing protein E3 ubiquitin ligase complex (SCF^Slim^-Cullin1). The relative amount of both Ci^R^ and Ci^A^ determines the transcriptional status of Hh-target genes, such as *ptc*, *dpp* and *engrailed* (*en*) [41–44].

In the developing *Drosophila* eye, while low levels of Hh signaling promotes cell shape changes associated with MF formation and concomitant *dpp* expression, high levels are required for the re-entry of the precursor cells in the cell cycle at the SMW and for the activation of *roadkill (rdx*) expression [45–48]. Rdx targets Ci^FL^ to full degradation through the recruitment of the Cullin3 (Cul3)-based E3 ligase complex [45,46]. Thus, posterior to the MF, high levels of Hh signaling attenuate its own activity by Rdx:Cul3 complex, allowing retinogenesis to occur properly [45,46]. Therefore, mutations that affect MF progression could be additional components of the machinery that regulates Hh signaling intensity and dynamics. Mutations in the retinal determination gene *dachshund (dac)* affect MF movement, without blocking differentiation [49]. *dac* expression depends on Hh signaling itself [25,50]. It localizes to all nuclei straddling the Ci^FL^-expressing domain, from the progenitor domain to the SMW, where differentiating PR cells start expressing *hh* (Fig 1B and 1C–1C´´). Therefore, high Dac levels coincide with the major neuronal differentiation and morphogenetic processes controlled by Hh signaling. Altogether, these results indicate that *dac* exhibits the traits required for being a candidate modulator of Hh signaling intensity. Here, we show that indeed *dac* potentiates Hh signaling in the MF by promoting Ci^FL^ accumulation and Ci^A^ activity downstream or in parallel of Fu. Our observations argue that this mechanism is absolutely required to promote proper retinogenesis by controlling the timing of MF formation, accurate specification of the founder PR cell and to trigger the Rdx-dependent negative feedback, which turns Hh signaling off posterior to the MF. Thus, Hh signaling potentiation by Dac allows the fast building up of signaling that is required for the swift processes associated with the moving retinal differentiation wave in *Drosophila*.

## Results

### *dac* controls the timing of MF formation and proper retinogenesis

To investigate a role of *dac* as a candidate modulator of Hh signaling, we reexamined the consequence of removing *dac* on MF-associated processes. Consistent with previous observations [49], all GFP-marked *dac-* clones larger than 6 cells straddling this region showed a delay in MF formation (Fig 1D, S1A and S1A´ Fig; n = 53). Accordingly, the onset of PR differentiation, detected by labeling with an antibody against the neuronal marker Elav, was also retarded in these clones (Fig 1E and 1E´, S1D and S1D´ Fig). This retardation was associated with a delay in the onset of *ato* expression and with an aberrant spacing of Ato-positive PR8 cells (Fig 1, compare 1G and 1G´ with 1F). These defects were not specific of any ommatidial cell type: in *dac*- clones posterior to the MF, we detected expression of the *hh-Z* enhancer trap, which marks PR2-5 cells (Fig 1H and 1H´), of the PR3/4 marker Spalt (Sal) (Fig 1I) and of the PR7 marker Prospero (Pros) (Fig 1J). However, the density of ommatidia (Fig 1E and 1E´, 1H and 1H´, S1D and S1D´ Fig) and the proper number of cell types per ommatidium were affected by the loss of *dac* function. For instance, some ommatidia only contained one Sal-expressing cell instead of two (yellow arrow in Fig 1I).

Concomitant with the delayed MF and differentiation onset, the SMW was also retarded and became asynchronous. Posterior to the SMW, *dac-* clones showed persistent reentry into the cell cycle, as detected by elevated expression of the G2/M CyclinB (CycB) (S2A and S2A´ Fig), increased number of cycling cells (S2B and S2B´, S2C and S2C´ Fig), as well as maintenance of the expression of the G1/S cyclin CyclinE (CycE) and loss of *dap* (S2D and S2D´ and S2E Fig). Taken together, we conclude that *dac* is required for three essential roles played by Hh signaling: MF movement, regulation of the cell cycle and proper retinogenesis.

### Dac potentiates Hh signaling

**Fig 2:**
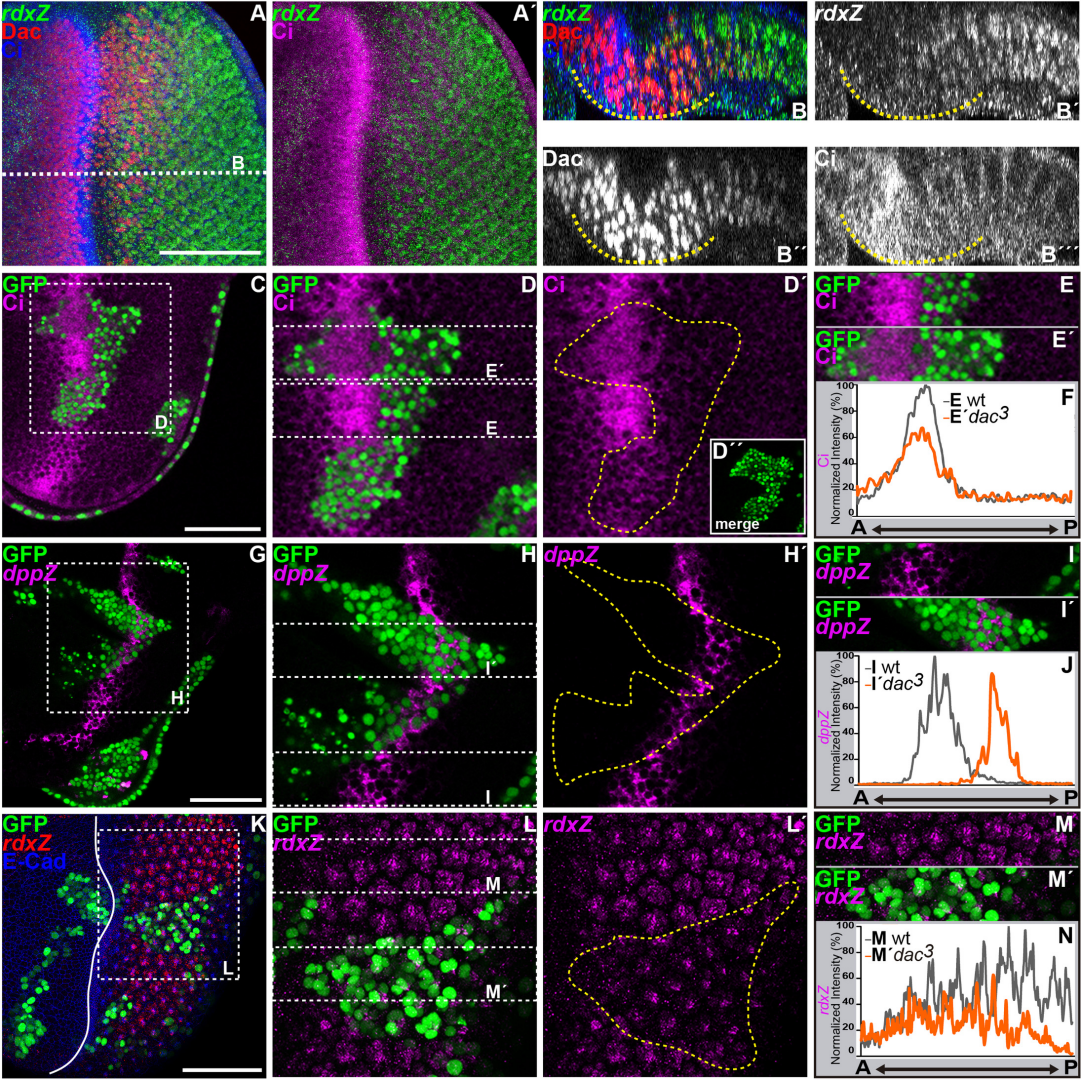
Loss of *dac* reduces Ci^FL^ and *dppZ* levels in the MF and fails to potentiate *rdxZ* posterior to the MF. All panels show L3 eye imaginal discs, except F,J,N. (A-B´´´) Standard confocal section (A-A´) and cross-section (B´´´) through the dashed white line in B of a wild type disc stained with (A-B´) anti-β-Galactosidase to detect *rdxZ* (green in A-A´, B and white in B´), anti-Dac (red in A,B and white in B´´) and anti-Ci (blue in A,B; magenta in A´ and white in B´´´). Dashed yellow lines in B-B´´´ outline the domain where Dac is expressed at higher levels. (C-E´, G-I´, K-M´) *dac*^*3*^ clones marked by the presence of GFP (green in C,D,D´´,E-E´,G,H,I-I´,K,L,M-M´) and outlined by yellow dashed lines in D´,H´ and L´. Discs are stained with (C-E´) anti-Ci (magenta), (G-I´,K-M´) anti-β-Galactosidase to reveal (G-I´) *dppZ* (magenta) or (K-M ´) *rdxZ* (red in K and magenta in L-M´) and (K) anti-E-Cad (blue in K). Dashed white squares in C,G,K delimit the area shown in D-D´´, H-H´, L-L´, respectively. D´´ shows a merge of GFP signal relative to D. (F,J,N) Profiles of the intensity signals along the anterior-posterior (AP) axis of wild type (grey line) or *dac*^*3*^ mutant clones (orange line) for (F) Ci (at the levels of E or E´, respectively), or (J) *dppZ* (at the levels of I or I´, respectively) or (N) *rdxZ* (at the levels of M or M´, respectively). E-E´, I-I´ and M-M´ are outlined by dashed white rectangles in D,H,L. Plain white line in K’ indicates the MF. Scale bars represent 50μm.

Downregulating the function of the Hh-signal transducer *smo* (*smo*^*3*^ clones) also caused a delay in MF movement (seen by E-Cadherin (E-Cad) higher signal intensity) and affected apical constriction of cells within the MF (S1B and S1B´ Fig). In addition, the density of ommatidia was reduced in *smo-* clones (S1E and S1E´ Fig). Thus, *smo-* and *dac-*mutant clones shared similar phenotypes (Fig 1D, 1E and 1E´ and S2A and S2B´, S2D and S2E´ Fig). In agreement with *dac* and *smo* being part of the same signaling pathway, *dac* synergized with *smo* in MF formation and PRs differentiation. All *smo*, *dac* double-mutant cells failed to undergo the cell shape changes associated with the MF (S1C and S1C´ Fig, n = 33 discs) and to differentiate PRs (S1F and S1F´ Fig, n = 20 discs). As *dac* was expressed in cells that accumulated Ci^FL^ at high levels in the MF and at low levels posterior to the MF where Ci promotes the transcription of the *rdxZ* reporter (Fig 1B–1C´´ and 2A–2B´´´), we next analyzed if *dac* affected Hh signaling. Strikingly, 67% of discs containing GFP-marked *dac-* clones straddling the MF showed reduced Ci^FL^ levels (Fig 2C–2F; n = 24 discs) and lower transcription of *dpp*, monitored by the transcriptional reporter *dppZ* (Fig 2G–2J; 64% of discs, n = 14). In addition, all *dac-* clones failed to trigger high levels of a *lacZ* enhancer trap insertion in the *rdx* gene *(rdx*Z) posterior to the MF (Fig 2K–2N; n = 9 discs). All these results indicate that *dac* is required for a full activation of the Hh signaling pathway.

**Fig 3:**
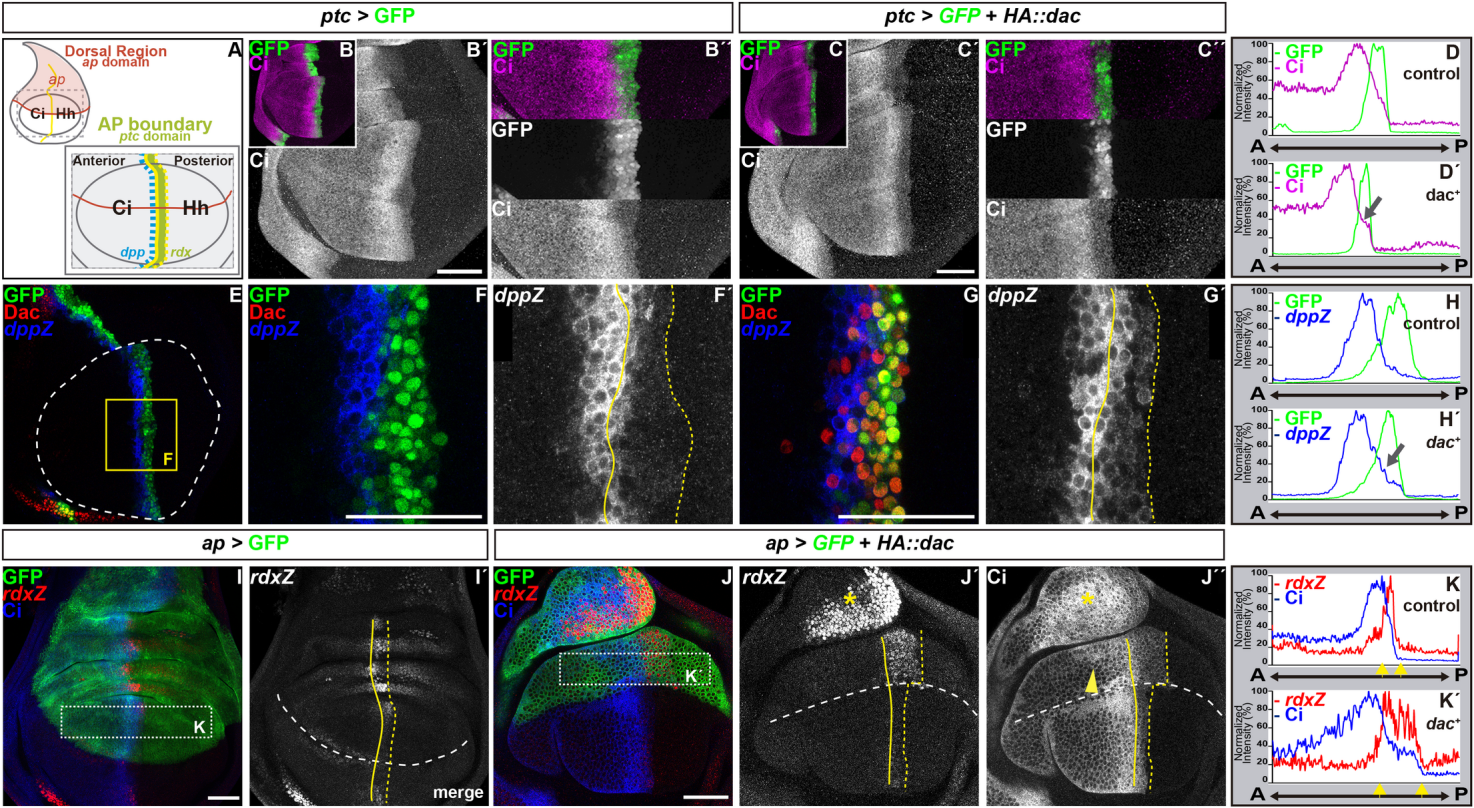
Expressing *dac* ectopically in the wing disc is sufficient to enhance Hh signaling. (A) Schematics of the whole wing disc (upper) and of the distal wing disc (lower) showing the expression domain of *ap* and of Hh pathway components. *ap and hh* are expressed in the dorsal (red) and posterior domain, respectively. Hh signals to the anterior domain leading to Ci^FL^ accumulation and activation of *rdx and ptc* in the anterior-posterior (AP) compartment boundary (green) and of *dpp* (blue). (B-C´´,E-G´, I-J´´) Standard confocal sections of L3 wing imaginal discs with anterior to the left and dorsal up. (B-C´´,E-G´) *ptc*-Gal4 driving the expression of UAS-*GFP* (B-B´´,E-F´) or UAS-*GFP* (C-C´´,G-G´) and UAS-*HA*::*dac*. (I-J´´) *ap*-Gal4 driving the expression of (I-I´) UAS-*GFP* or (J-J´´) UAS-*GFP* and UAS-*HA*::*dac*. Discs are stained with (B-C´´) anti-Ci (magenta in B,C, upper panel in B´´ and C´´; white in B´,C´, bottom panel in B´´ and C´´) or (E-G´) anti-Dac (red in E,F,G) and anti-β-Galactosidase to reveal *dppZ* (blue in E,F,G and white in F´,G´) or (I-J´´) anti-Ci (blue in I,J and white in J´´) and anti-β-Galactosidase to reveal *rdxZ (*red in I-J and white in I´,J´). B´´ and C´´ are magnification of B and C, respectively. (D-D´) Profiles of the GFP (green lines) or Ci (magenta lines) intensity signals across the AP axis in B´´ and C´´, respectively. (H-H´) Profiles of the GFP (green lines) and *dppZ* (blue lines) intensity signals across the AP axis in F and G, respectively. The arrows in D´ and H´ highlight the accumulation of Ci and *dppZ* in the *ptc>dac*-expressing domain, respectively. (K-K´) Profiles of the Ci (blue lines) and *rdxZ* (red lines) intensity signals across the AP axis in the dorsal areal delimited by dashed line in I and J, respectively. Yellow arrows in K and K´ indicate the position of the AP compartment boundary. The plain and dashed yellow lines in F´,G´,I´ and J´-J´´ delimited the AP compartment boundaries. The white dashed line in E outlines the distal wing blade. F-F´ is a magnification of the yellow square in E. The white dashed line in I´,J´-J´´ indicate the dorsal-ventral boundaries. The yellow arrowhead in J´´ indicates the accumulation of Ci in the dorsal, anterior blade. The yellow asterisks in J´,J´´ indicate the upregulation of *rdxZ* (J´) and the accumulation of Ci (J´´) in the proximal dorsal domain. Scale bars represent 50μm.

To determine if Dac is sufficient to potentiate Hh signaling, we analyzed the effect of overexpressing *dac* on Hh signaling activity in the wing imaginal disc, where endogenous Dac protein is expressed only in a few restricted patches [49]. In this tissue, Hh produced in the posterior (P) compartment signals to the anterior (A) compartment (Fig 3A). Thus, cells along the AP boundary compartment receive maximal Hh signaling, leading to the activation of *rdx* expression and consequently signaling attenuation through Rdx:Cul3-mediated Ci^FL^ degradation [45,46]. At these signaling levels, immediately adjacent to the P compartment, *ptc* expression is induced [41–44]. Next to this domain and further away from the AP boundary *dpp* is expressed [41–44]. Therefore, if *dac* potentiates Hh signaling, we expected that its expression along the AP boundary should enhance signaling levels and allow *dpp* transcription. Although overexpressing *dac (HA*::*dac)* along the AP boundary compartment using a *ptc-Gal4* driver (Fig 3A) promoted Ci^FL^ accumulation (Fig 3, compare 3C–3C´´ with 3B–3B´´ and 3D´ with 3D) and increased expression of *dppZ* (Fig 3, compare 3G and 3G´ with 3E, 3F and 3F´ and 3H´ with 3H), these effects were relatively modest. Overactivation of Hh signaling would also be expected to potentiate *rdx* transcription, which would limit Ci^FL^ accumulation and Hh signaling activity. In agreement with this, overexpressing *HA*::*dac* in the dorsal compartment using the *apterous-Gal4 (ap-*Gal4) driver (Fig 3A) upregulated *rdxZ* expression in 85% of discs analyzed (n = 20) and drastically extended the Ci^FL^-expression domain in the dorsal wing disc cells closed to the AP boundary in 82% of cases (n = 17) (Fig 3, compare 3J–J´´ with 3I and 3I´ and 3K´ with 3K). Taken together, we conclude that *dac* is necessary and sufficient to potentiate Hh signaling activity by promoting Ci^FL^ accumulation and the activation of Hh target genes.

### *dac* is required for Hh signaling downregulation and for ‘furrow state’ exit

**Fig 4:**
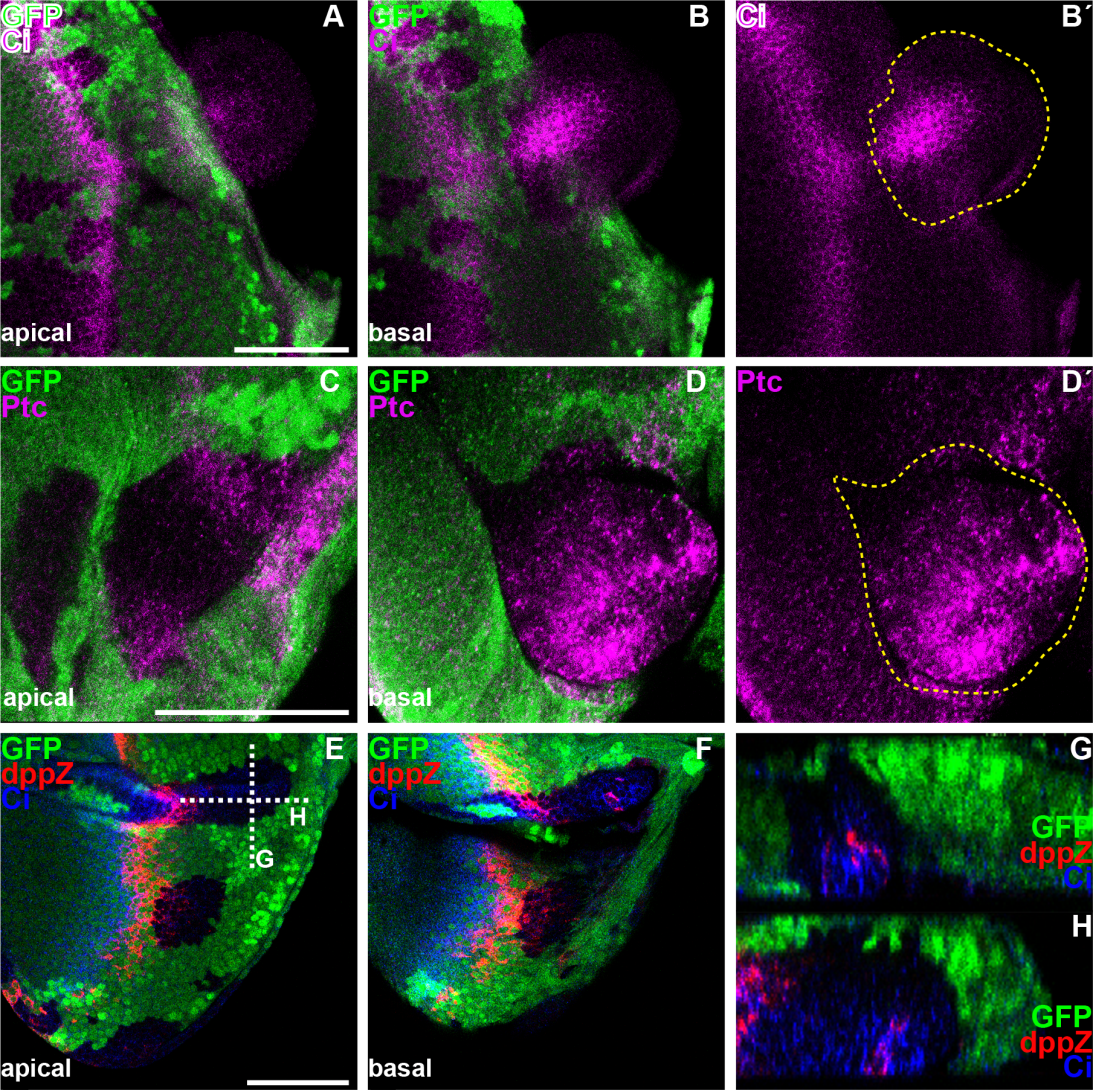
Loss of *dac* function leads to the persistence of Hh target genes expression posterior to the MF. All panels show L3 eye imaginal discs, containing *dac*^*3*^ clones marked by the absence of GFP (green in A,B,C,D,E-H) and outlined by yellow dashed lines in B’ and D’. A and B-B´ or C and D-D´ or E and F are apical (A,C,E) or basal (B-B´, D-D´,F) standard confocal sections of the same discs. (G-H) Cross-section through the disc epithelium at the level of the white dashed lines in E. Discs are stained with (A-B´) anti-Ci (magenta) or (C-D´) anti-Ptc (magenta) or (E-G´) anti-Ci (blue) and anti-β-Galactosidase (red) to reveal *dppZ*. Note that *dac* mutant tissues appear to drop out from the disc epithelium. Scale bars represent 50μm.

**Fig 5:**
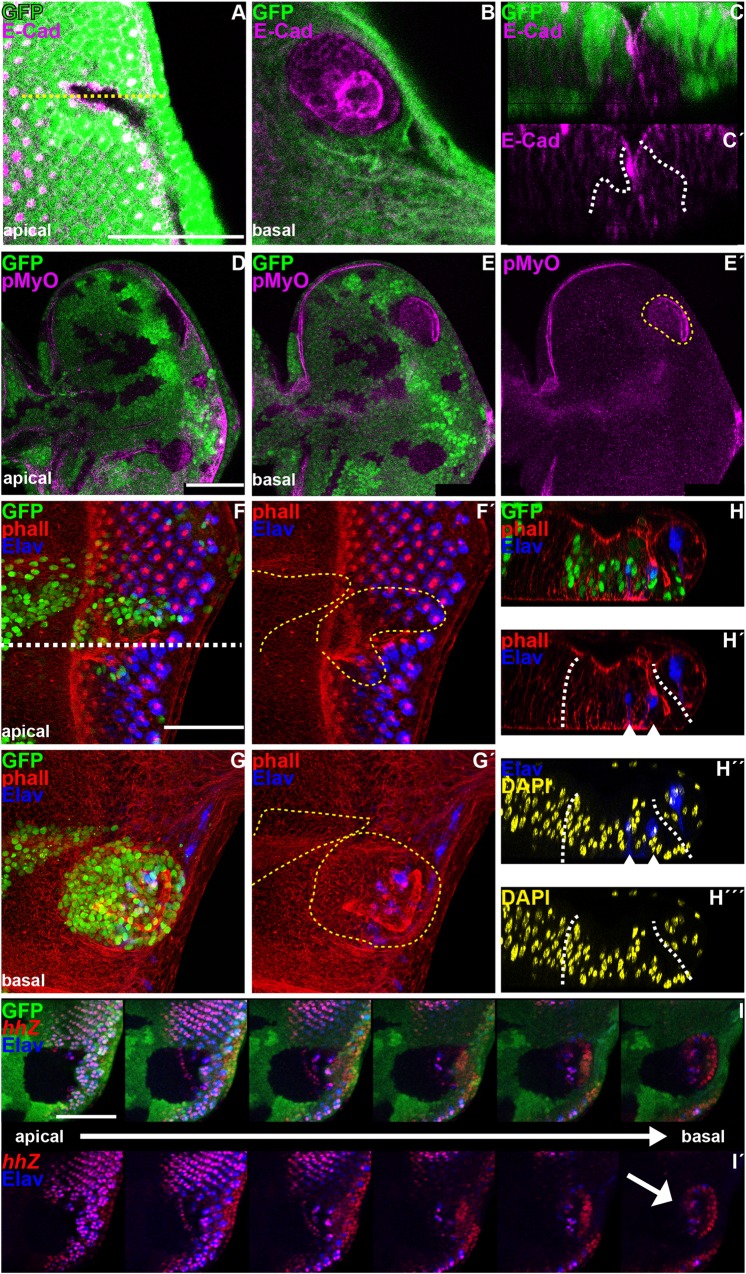
Loss of *dac* maintains a ‘furrow state’ posterior to the MF. All panels show L3 eye imaginal discs, containing *dac*^*3*^ clones marked by the (A-E´ and I-I´) absence or (F-H´´´) presence of GFP (green in A, B, C, D, E, F, H, G and I) and outlined by dashed lines in C´, E’, F´ and H´-H´´. A and B or D and E-E´ or F-F´ and G-G´ are apical (A,D,F-F´) or basal (B, E-E´,G-G´) standard confocal sections of the same discs. I and I´ show subsequent standard confocal sections from apical to basal. (C-C´,H-H´´´) Cross-sections through the disc epithelium at the level of the yellow dashed lines in A or F, respectively. Discs are stained with (A-C´) anti-E-Cad (magenta) or (D-E´) anti-pMLC (magenta) or (F-H´´´) anti-Elav (blue) and phalloidin (red in F,F´,G,G´,H and H´) and DAPI (yellow in H´´ and H´´´) or (I-I´) anti-Elav (blue) and anti-β-Galactosidase (red) to reveal *hhZ*. Arrows in H´,H´´ and I´ indicate the basal localization of PRs in *dac*- clones. A minimum of 24 clones in internal region of the disc primordium was observed to maintain a “furrow state” posterior to the MF. Scale bars represent 50μm.

The Hh pathway has built-in a negative feedback that serves to attenuate signaling following maximal activation. This feedback rests on the activation of *rdx* by high signal levels. Once expressed, Rdx drives a Cul3-dependent Ci^FL^ degradation posterior to the MF thus allowing the exit from the furrow state [45,46]. We therefore tested if the loss of *dac* function induces the persistence of Hh signaling posterior to the MF. Indeed, some *dac-* clones located in internal region of the disc primordium showed ectopic expression of Ci^FL^ (Fig 4B and 4B´) and of the Hh-target genes Ptc (Fig 4D and 4D´) and *dpp*Z (Fig 4F–4H). Strikingly, all these clones dropped basally (Fig 4A, 4C and 4E). As sustained exposure to Hh signaling promotes MyOII-dependent cell ingression and groove formation [32,33], we analyzed the shape of *dac-* clones posterior to the MF using the apical marker E-Cad. We confirmed that the disappearance of *dac-* cells from the apical surface (Fig 5A) did not result from a loss of cell polarity, as *dac-* cells maintained E-Cad expression apically (Fig 5B). However, cross section through the eye disc showed that *dac-* clones ingressed within the epithelium, forming grooves (Fig 5C and 5C´). In addition, these clones accumulated activated MyOII, detected by phospho-Myosin Light Chain (pMLC) antibody at the apical surface of ingressed clones (Fig 5D, 5E and 5E´). These drastic changes in cell shape were associated with the presence of PR nuclei, expressing Elav and *hh*Z that were still localized close to the apical cell surface but appeared on basal focal planes compared to control GFP-positive neighboring nuclei (Fig 5F and 5F´ to 5I and 5I´). *dac-* clones spanning the disc margin that delayed MF progression and PRs differentiation also contained higher Ptc levels (S3A and S3A´´ Fig). However, those in which MF initiation and retinogenesis were compromised [49] showed reduced Ptc expression (S3B and S3B´ Fig). Thus, *dac* is required for the swift dynamic changes in Hh signaling associated to the passing MF. In its absence, Hh target genes activation and Hh-regulated processes suffer a general delay. Interestingly, one of the consequences is that Hh signaling persists for longer, as its attenuation mediated by *rdx* is also delayed.

### *dac* is required downstream of *fu* to promote Ci accumulation

**Fig 6:**
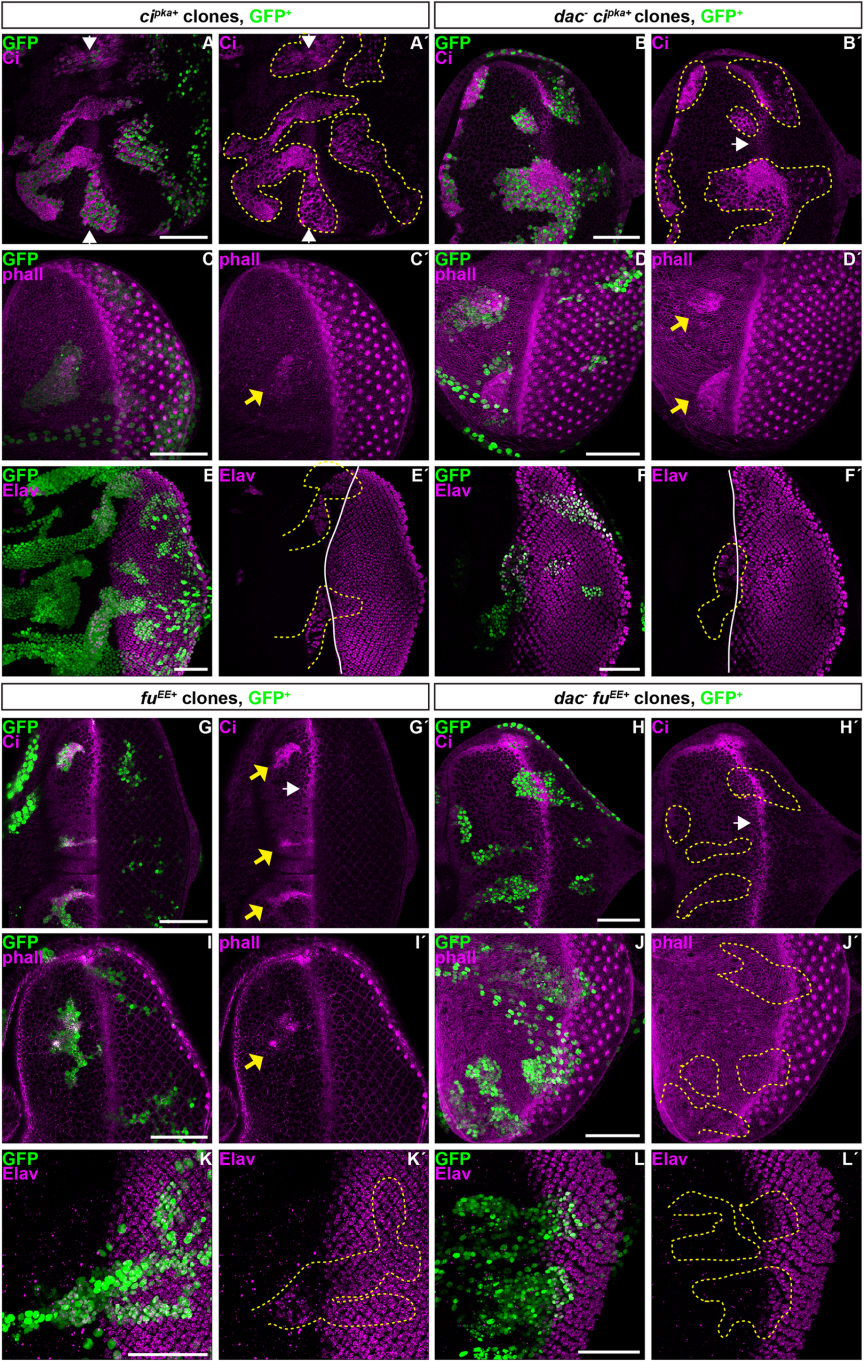
Removing Dac function suppresses the activity of Fu^EE^, but not of Ci^pka^. All panels show standard confocal sections of L3 eye imaginal discs, containing clones labeled with GFP (green in A,B,C,D,E,F,G,H,I,J,K,L) and outlined by dashed lines in A´,B´,E´,F´,H´,J´,K´,L´ and containing clones (A-A´,C-C´,E-E´) expressing UAS-*ci*^*pka*^ and (B-B´,D-D´,F-F´) mutant for *dac*^*3*^ or (G-G´,I-I´,K-K´) expressing UAS-*fu*^*EE*^ and (H-H´,J-J´,L-L´) mutant for *dac*^*3*^. Discs are stained with (A-B´,G-H´) anti-Ci (magenta) or (C-D´,I-J´) phalloidin (magenta) to outline cell shape or (E-F´,K-L´) anti-Elav (magenta)White arrows in B´,G´ and H´ indicate Ci^FL^ accumulation in wild type cells in the MF. Yellow arrows in G´ indicate Ci^FL^ accumulation anterior to the MF. Yellow arrows in C´, D´ and I´ highlight increased levels of phalloidin staining, reminiscent to apical contraction of the MF. Arrows in A-A´ and solid lines in E´ and F´ indicate MF position. Scale bars represent 50μm.

To understand how *dac* promotes Ci^FL^ accumulation, we analyzed the requirement for *dac* to transduce Hh signaling in cells expressing constitutive active forms of Hh pathway components. We first expressed in clones a form of *ci* insensitive to phosphorylation by PKA *(ci*^*pka+*^*)*. Consistent with previous observations, *ci*^*pka+*^ clones promoted precocious differentiation anterior to the MF, where cells express *dac* endogenously [20,51–56]. All *ci*^*pka+*^ clones accumulated Ci^FL^ (Fig 6A and 6A´; n = 7 discs), while 89% displayed an enrichment of F-actin, reminiscent to the apical cell constriction in the MF (Fig 6C and 6C´; n = 27 discs) and 62% formed ectopic PRs (Fig 6E and 6E´; n = 8 discs). However, posterior to the MF, where Ci^FL^ degradation is independent of PKA [57], Ci^pka+^ accumulation was reduced when compared to clones located in the MF and anterior to the MF (Fig 6A and 6A´). Thus, Ci^pka+^ may suffer degradation in this domain. Removing *dac* function did not affect the ability of clones expressing ectopic *ci*^*pka+*^ to accumulate Ci^FL^ (Fig 6B and 6B´; 100% of discs, n = 10), F-actin (Fig 6D and 6D´; 95% of discs, n = 22) or differentiate ectopic PRs (Fig 6F and 6F´; 22% of discs, n = 12) anterior to the MF. Therefore, *dac* acts upstream of Ci^FL^.

We next investigated if *dac* was required for the activity of the upstream Ci regulator Fused (Fu). Overexpressing a constitutive active form of *fu* (*fu*^*EE+*^) anterior to the MF also triggered Ci^FL^ accumulation in 93% of discs with clones (Fig 6G and 6G´, n = 27 discs), an enrichment of F-actin or E-Cad, reminiscent to the apical cell constriction in the MF in 85% of cases (Fig 6I and 6I´; n = 13 discs), and ectopic PR differentiation in 11% of discs with clones (Fig 6K and 6K´; n = 27 discs). In contrast, when these clones were also mutant for *dac*, the accumulation of Ci^FL^ (Fig 6H and 6H´; 36% of discs, n = 11) and of F-actin (Fig 6J and 6J´; 41% of discs, n = 27) was severely reduced. In addition, these clones were no longer able to differentiate ectopic PRs (Fig 6L and 6L´, n = 6). Further, overexpressing *fu*^*EE+*^ did not rescue the MF delay of *dac*-mutant tissue (Fig 6H and 6H´ and 6J and 6J´)). Thus, *dac* potentiates Hh signaling by promoting Ci^FL^ accumulation downstream of, or parallel to *fu*.

*dac* encodes a nuclear protein, which has been shown to bind double-stranded nucleic acids [58] and to activate transcription of a reporter gene in yeast [59]. We therefore tested, using a genome-wide ChIP-seq approach, the possibility that Dac regulated the expression of some of the components along the Hh signaling pathway (see Materials and Methods). We analyzed specifically ChIP peaks in distal regions (i.e. excluding those falling in 5’UTRs and 1kb upstream of transcription start sites). Within the ChIP peaks, we identified a set of 352 distal regions in which we found significantly enriched an A-rich motif. This motif is similar to the DNA binding motif identified previously for the human DACH by protein structure, in-silico and ChIP-seq analyses (S4 Fig, [60]). This fact indicated that the set of 352 ChIP peaks were likely directly bound by Dac. However, among the nearby genes (S4 Fig), we could not identify any of the major components of the Hh signaling pathway (including *hh* itself plus *smo*, *PKA*, *CKI*, *ci*, *cullin1*, *Slimb*, *skp-1*, and *cul3*). This result suggested that the effect of *dac* on the activity of the Hh pathway was unlikely to be mediated by the transcriptional regulation of these signaling components. To test experimentally this point, we generated *dac-* clones and asked whether *dac* loss affected the expression of the *hhZ* and *ciZ* enhancer traps, which serve as transcriptional reporters. Although *dac* mutant clones located in internal region of the eye primordium appeared to contain reduced *hhZ* expression (Fig 1H and 1H´), this effect likely resulted from a reduction in the number of PR cells per ommatidia in *dac-* mutant tissue, as PR *hhZ* levels were not different than in wild type cells. Similarly, in clones straddling the MF, the absence of *dac* function delayed *hhZ* expression, but appeared normally as PR cells gained ELAV signal (S5A and S5A´´´ Fig). In addition, *dac* was neither necessary nor sufficient to control *ci* transcription, as *ciZ* expression was not affected in *dac* mutant in the eye discs (S5B and S5C´ Fig) or in wing discs overexpressing *dac* using the *ptc-*Gal4 driver (S5 Fig, compare S5H to S5J with S5D to S5F and S5K with S5G). We conclude that *dac* does not promote Ci^FL^ accumulation downstream of, or parallel to *fu* by affecting *hh* or *ci* expression and, most likely, neither affecting the transcription of other major pathway components.

## Discussion

The differentiation of the retina in *Drosophila* occurs both very fast and with high precision: every 2 hours, one new column of assembled ommatidia is added to the developing eye [61]. This phenomenon requires the coordination of gene expression, cell cycle and tissue morphogenesis. All these processes depend critically on the dynamics of Hh signaling. In this report, we show that Dac is an essential element in this dynamics. Dac potentiates Hh signaling in the MF, upstream of Ci and downstream of, or in parallel to Fu. By doing so, *dac* ensures proper retinogenesis by controlling the timing of MF formation, the accuracy of cell cycle control, the tissue changes associated to the MF, the correct specification of the founder PR8 cell and the attenuation of Hh signaling posterior to the MF, which allows the progression of the differentiation wave.

### *dac* potentiates Hh signaling

Our observations demonstrate that *dac* is required to strengthen Hh signaling. First, the absence of *dac* function recapitulates phenotypically a reduction of Hh signaling. Removing *smo* or *dac* function delays MF progression (Figs 1 and 2, S1 Fig, [23,28–31,49]) and the re-entry in S-phase in the SMW (S2 Fig; [25]). In addition, loss of *dac* (Fig 1) or *smo* [23,29,31] results in reduced *ato* expression ahead of the MF and affects the restriction of *ato*, first in proneural clusters and then in single PR8 posterior to the MF. Furthermore, neither loss of *dac* (Fig 1), nor *smo* [31,35] affects ommatidial cell fate. The cause of the reduced number of ommatidia and variable number of cells per ommatidium in *dac* mutant clones (Fig 1) is not totally clear. It could result from the effects in *ato* expression, including its abnormal spacing posterior to the MF and the singling of the ommatidial founder PR8 [62–67]; it could also arise from alterations in cell cycle control, as we detect persistent cell cycling posterior to the MF, which may affect cell recruitment into the ommatidium [25,36]; or from a combination of both. Second, *smo* and *dac* synergize to promote MF progression and PR differentiation (S1 Fig). Accordingly, removing one copy of *hh* enhances the *dac* mutant eye phenotype and fully suppresses PRs differentiation of *dac* mutant clones located in internal regions of the disc primordium [49]. Third, *dac* mutant clones show reduced levels of Ci^FL^ and lower expression of the Hh target gene *dpp* in the MF and *rdx* posterior to the MF (Fig 2). In addition, *ptc* expression, another Hh target, is reduced in marginal *dac* mutant clones that fail to differentiate photoreceptor cells (S3 Fig). Conversely, expressing *dac* ectopically in the wing disc is sufficient to enhance Ci^FL^ accumulation and *rdx* expression, and to induce *dpp* in the domain immediately adjacent to the compartment boundary (Fig 3).

### How does *dac* regulate Hh signaling?

Our observations argue that *dac* potentiates Hh signaling by promoting Ci^FL^ accumulation downstream of, or in parallel to Fu. First, loss of *dac* reduces Ci^FL^ levels in the MF (Fig 2), while expressing *dac* ectopically in the wing disc has the opposite effect (Fig 3). Second, an activated form of Fu (Fu^EE+^), which inhibits Ci processing (into Ci^R^) and promotes Ci^FL^/Ci^A^ accumulation [68], requires *dac* function for this accumulation of Ci^FL^ and to induce MF-like features and precocious PR differentiation anterior to the MF. In contrast, removing *dac* function has no major effect on the ability of a PKA-insensitive form of Ci (Ci^pka+^) to trigger features associated to ectopic Hh signaling in this domain (Fig 6). The precise molecular mechanism by which Dac affects this step along the Hh signaling pathway is unknown at the moment. *dac* encodes a nuclear protein, which has been shown to bind double-stranded nucleic acids [58] and to activate transcription of a reporter gene in yeast [59]. Our functional and ChIP-seq experiments indicate that *hh* and *ci* are not direct transcriptional targets of Dac. In addition, the ChIP-seq data suggest that neither are any of the major pathway components of the Hh pathway (S4 Fig)–although without further studies this possibility cannot be ruled out completely. This would shift the control of the Hh signaling activity to other Dac targets, which would exert this control directly or indirectly. Another, not mutually exclusive possibility is that Dac collaborates with Ci^A^ in enhancing the expression of Ci^A^-dependent target genes, such as *rdx* or *ptc* (Figs 2 and 3). Although we have previously shown that Dac inhibits the transcriptional ability of the Homothorax/Yorkie (Hth/Yki) complex [69], Dac may act as an activator or repressor depending on the cellular context [70]. Therefore, Dac may contribute to the transcriptional activity of Ci^A^ promoting the expression of target genes responsible for proneural fate acquisition and differentiation. In agreement with this hypothesis, using the bioinformatic tool Clover [71], with standard parameters, we also found, besides the Dac motif, the Gli/Ci consensus binding motif significantly enriched (p<0.001) in the distal Dac-ChIP-peaks (S4 Fig), suggesting that Dac might indeed collaborate with Ci^A^ to enhancer expression of its targets. In addition, we cannot exclude other mechanisms of Dac action independent of its role in transcriptional regulation. In fact, although it was not reported in *Drosophila* tissues, the human Dac homologue DACH1 presents both nuclear and cytoplasmic localization in different tissues [72–75]. Moreover, DACH1 localization shifts from the nucleus in normal tissue to the cytoplasm in ovarian cancer [72]. Dac/DACH1 might be involved in the control of Hh signaling pathway in a subcellular localization manner. Further studies are required to elucidate the mechanism by which Dac affects Ci^FL^ accumulation and consequently potentiates Hh signaling.

### *dac*¸ an orchestrator of eye development that promotes Hh signaling dynamics

**Fig 7:**
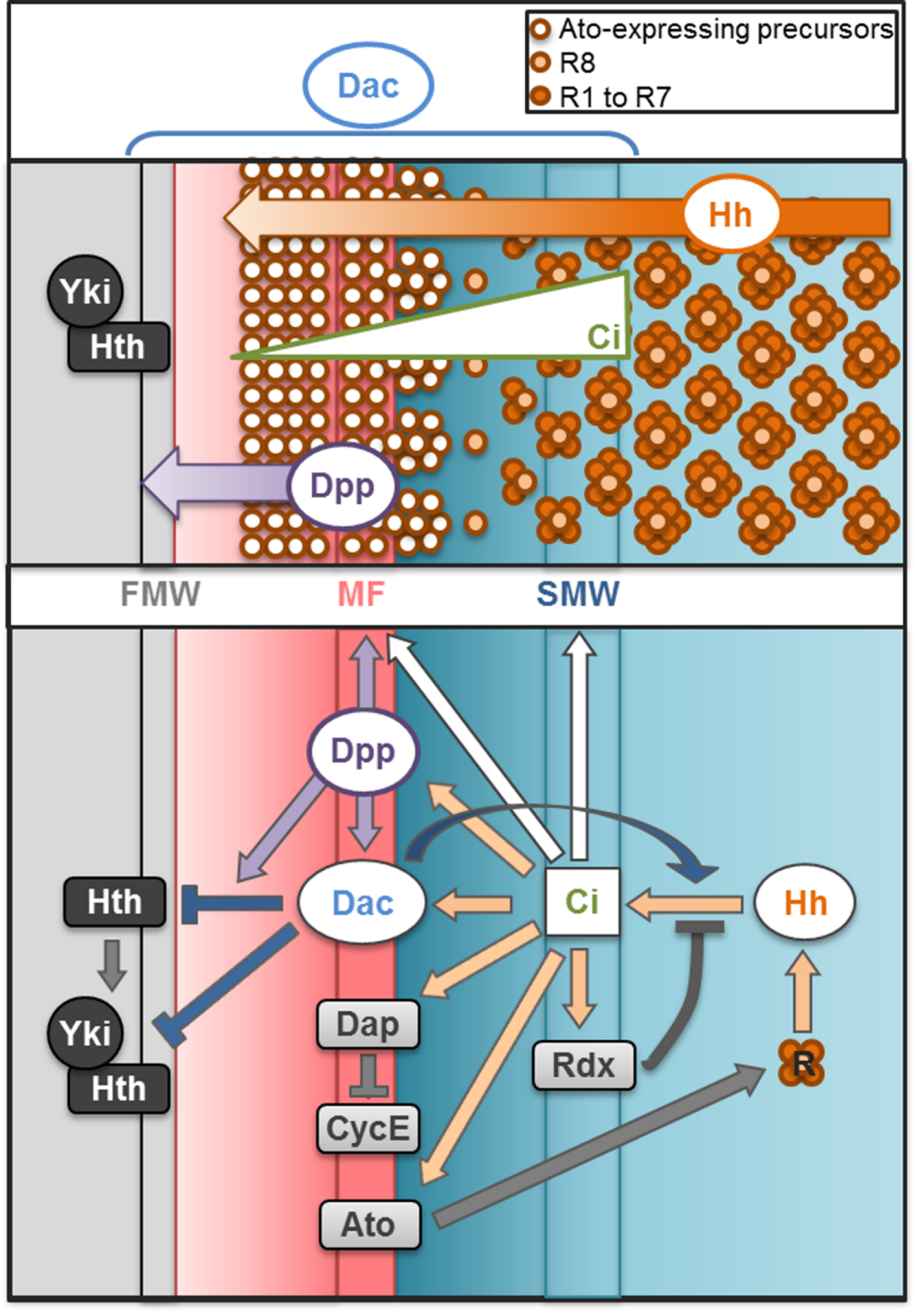
Model by which Dac orchestrates retinogenesis. Schematic of the L3 eye disc spanning from the anterior proliferating progenitors (left) to the differentiated photoreceptors (right), showing the expression domain and activity of Dac, Hh, Ci, Dpp, and Yki/Hth (upper panel) and their molecular interactions to coordinate proliferation and differentiation of the retina (lower panel). Hh expressed by the differentiating PRs diffuses short-range, promoting a gradient of Ci activity. High levels of Ci promote the synchronized re-entrance in the cell cycle in the SMW [25,34–36] and turns off its own signaling pathway via *rdx* expression [45–48]. Anterior to this domain, low Hh signaling initiates the expression of *ato*, which promotes the acquisition of a proneural fate [18–22]. In addition, in this domain, Hh signaling upregulates *dap*, which keeps precursor cells in G1-phase by inhibiting CycE [24–27], and activates *dpp* expression [12]. Together, Hh and Dpp induce MF formation and high *dac* expression [50]. In turn Dac, whose expression extends from the FMW to the SMW, straddling the MF, ensures the transition from proliferation to G1 arrest state by inhibiting the transcriptional activity of the Yki-Hth complex in the FMW [69]. Moreover, together with Dpp, Dac represses Hth expression [69]. Finally, we show here that Dac potentiates Hh signaling in the MF, upstream of Ci. This positive feedback loop is essential to control all Hh functions, including the timing of MF formation, the accuracy of cell cycle control, the tissue changes associated to the MF, the correct specification of the founder PR8 cell and the attenuation of Hh signaling posterior to the MF, which allows the progression of the differentiation wave.

In the developing eye, *dac* lies downstream of *eye absent* (*eya)* and the *dpp* signaling pathway [50,76–78] where it regulates multiple events that together coordinate cell proliferation and differentiation in time and space. We demonstrate here that *dac* potentiates Hh signaling. This together with *dac’s* role in the transition from proliferating progenitor cells to committed precursor cells together with Dpp signal [69] can account for the pleiotropic and essential roles played by *dac* (Fig 7). Retinogenesis starts with the formation of the MF and the triggering of two major Hh targets: *dpp* and *ato*. The weakening of Hh signaling together with the reduced *dpp* transcription can account for MF delay, as both signals are required for this morphogenetic event [18,19,23,28–31]. Right at the MF and immediately posterior to it, the cell cycle is tightly regulated. We observe that the expression of the p21/p27 Cdk inhibitor *dap* is lost in *dac-* cells (S2E Fig) and that this is accompanied by persistent cycling beyond the SMW (S2A and S2C´ Fig). This cell cycle misregulation, together with the aberrant restriction of *ato* expression, is the likely cause of the abnormal retinogenesis in *dac-*mutant tissues that includes ommatidia with variable number of cells. Posterior to the MF, high Hh signaling levels activates the expression of Rdx, which, together with Cul3, targets Ci^FL^ to full proteasomal degradation. In doing so, Hh signal becomes attenuated, and this attenuation allows cells to exit the furrowed state. In fact, *dac-*mutant clones posterior to the MF often remain as apically constricted inpouchings of the disc epithelium with high levels of P-MyOII, accumulated Ci^FL^ and expression of Ci^A^ target genes (Figs 4 and 5).

This report, together with our previous study [69], places *dac* as an essential regulator of retinal development, controlling the transitions from proliferating progenitor cells to committed precursor cells first [69] and then from precursors to differentiating retinal cells (this work). The *dac* expression profile spans the regions of the eye disc where it exerts its functions: the increasing Dac expression in precursor cells approaching the MF ensures that cells transit from proliferation to G1 arrest, while peak levels in the MF secure proper retinogenesis by potentiating Hh signaling here. High *dac* expression in the MF in addition to its downregulation in differentiating photoreceptors could both contribute to turn off Hh signaling in this domain. This could be achieved, at least in part, by inducing the activation of a negative feedback via *rdx* expression and by limiting Ci^FL^ accumulation downstream or in parallel to Fu, respectively. Whether this function is also carried out by the vertebrate *dac* homologues, the DACH1 and DACH2 genes, awaits further investigation.

## Materials and Methods

### Fly strains and genetics

Fly stocks used were *dac*^*3*^, *smo*^*3*^, *hh*^*P30*^ *(hhZ)*, *rdx*^*03477*^ *(rdxZ), P{dpp-lacZ.Exel.2}3 (dpp-Z),ciZ [79],* UAS-*HA*:*dac*^*F*^ [80], *UAS-mCD8GFP*, UAS-*mCherry (TRIP #35787)*, UAS-*fu*^EE^ [81–83], UAS-*Ci*^*pka*^ [82], *ptc-*Gal4 [84], *ap-*Gal4 [85]. Mutant clones for *dac*^*3*^ marked by the absence of GFP were generated through mitotic recombination [86]. The MARCM technique [87] was used to induce clones marked by the presence of GFP, mutant for *dac*^*3*^ or *smo*^*3*^ *or smo*^*3*^ *and dac*^*3*^ *or* expressing UAS-*fu*^*EE*^ or UAS-*ci*^*pka*^ *or* mutant for *dac*^*3*^ and expressing UAS-*fu*^*EE*^ or UAS-*ci*^*pka*^. Larvae were heat-shocked for 1 hour at 37°C between 48 and 72h after egg laying. Gain of function experiments using UAS-*HA*:*dac*^*F*^ were performed using the *ptc-*Gal4 or *ap-*Gal4 driver that drives expression in the AP boundary compartment or dorsal region of the wing disc, respectively. Crosses carrying UAS-*HA*:*dac*^*F*^ and the corresponding controls were raised at 18°C, while others were raised at 25°C.

### Immunohistochemistry

Imaginal discs were dissected and fixed according to standard protocols. Primary antibodies used were mouse anti-Dac (1:100; mAbdac2.3, DSHB); mouse anti-CycB (1:25; F2F4, DSHB); mouse anti-β-Galactosidase (1:200; Z378B, Promega), rabbit anti-β-Galactosidase (1:1000; 55976, Cappel); rat anti-E-Cad (1:50; DCAD2, DSHB); guinea pig anti-CycE (1:1000; gift from T. Orr-Weaver, Whitehead Institute, Cambridge, USA); rabbit anti-Ato (1:5000; [22]); rabbit anti-pMLC (1:10; 36715, Cell Signaling), which reveals pMyOII; rat anti-Elav (1:1000; 7E8A10, DSHB); mouse anti-Elav (1:100; 9F8A9, DSHB); guinea pig anti-Sens (1:1000; [88]); guinea pig anti-Pros (1:25; [89]); rabbit anti-Sal (1:200; [90]); rabbit anti-PH3 (1:200; 9701, Cell Signaling); mouse anti-Ptc (1:100; *Drosophila* Ptc (Apa1), DSHB); rat anti-Ci (1:10; 2A1, DSHB). Rhodamine-conjugated (Sigma) and C660–conjugated Phalloidin (Biotium) were used at a concentration of 0.3 μM and 5U/ml, respectively. DAPI was used at a concentration of 1ng/ml. Fluorescently labeled secondary antibodies were from Jackson Immunoresearch, (1:200). Imaging was carried out on Leica SP2 or SP5 confocal microscopy set ups. Plot profiles of fluorescent intensity were obtained using an NIH ImageJ program [91]. Fluorescent intensities were normalized to the maximum intensity for each channel.

### *In situ* hybridization

Anti-mouse-HRP (Sigma) was used for immunoperoxidase staining. Digoxigen labelled *dap* RNA probe (Roche) was produced from the cDNA clone LP07247 (BDGP). For BrdU incorporation assay, eye-antennal discs were dissected and incubated in 10μM BrdU in PBS for 30min. BrdU was detected with an anti-BrdU antibody (1:400; Roche) after treatment with DNase.

### Dac-ChIP Seq

Wandering 3^rd^ instar larvae (Dac:GFP, Bloomington stock 42269) were dissected in cold PBS and imaginal discs were fixed with formaldehyde for 25 minutes. Chromatin was fragmented by sonication till it reached an average size of 500 bp. 20 μl of protein A/G magnetic beads (Merck, Millipore) was added to pre-clean the samples. The anti-GFP Ab (ab290, Abcam) was added to a fixed chromatin aliquot and incubated at 4°C overnight. Immunocomplexes were recovered by adding protein A/G magnetic beads to the sample and incubating for 3 hours at 4°C. Beads were resuspended in elution buffer, RNase was added to the immunoprecipitated chromatin and incubated for 30 minutes at 37°C. ChIP libraries were prepared with the Truseq DNA library prep kit (Illumina) and the samples were sequenced on a HiSeq 2000 (Illumina). The reads were cleaned using fastq-mcf and mapped with bowtie2 to the *Drosophila melanogaster* genome (Flybase version 5). Dac-ChIP peaks (minus input) were called using macs2 (dac-ChIP-vs-ChIP_Input_peaks.bed). From this bed file all regions lying in a 5’UTR or 1kb upstream of a TSS were removed using bedtools intersectBed, to retain only distal Dac-ChIP-peaks (dac-ChIP-vs-ChIP_Input_peaks_not-in-5UTR_not-in-1kb-up.bed). The distal Dac-ChIP-peaks were loaded into i-cistarget [92], a motif enrichment tool, to obtain a final table (S4 Fig) of potential Dac targets. Access to the whole dataset can be found in the GEO database with the accession number GSE82151 (http://www.ncbi.nlm.nih.gov/geo/query/acc.cgi?acc=GSE82151), used as reference for all subsequent manuscripts referring to these data.

## Supporting Information

S1 Fig Removing *dac* function abrogates the ability of *smo* mutant clones to form a MF and differentiate PR cells. All panels show standard confocal sections of L3 eye imaginal discs, containing (A-A´´,D-D´) *dac*^*3*^ or (B-B´,E-E´) *smo*^*3*^ single mutant clones or (C-C´,F-F´) *smo*^*3*^, *dac*^*3*^ double mutant clones marked by the presence of GFP (green in A, A´´,B,C,D,E,F) and outlined by dashed line (in A´, A´´,B´,C´,D´,E´,F´). Discs are stained with (A-B´) anti-E-Cad (magenta) or (C-C´) phalloidin (magenta) to outline cell shape or with (D-F’) anti-Elav (magenta) to label differentiating PRs. A´´ corresponds to a merge between the GFP signal in A and more basal sections. Note that while loss of *smo* function has little effect on MF formation (arrow in B´) and PR differentiation, loss of both *smo* and *dac* function completely disrupts MF formation (arrows in C´) and PR differentiation. Scale bars represent 50μm.
(TIF)

S2 Fig Loss of *dac* function delays the SMW. All panels show (A-D´) standard confocal sections or (E) standard brightfield image of L3 eye discs, containing *dac*^*3*^ mutant clones marked by the absence of GFP (green in A,B,C,D and brown in E) and outlined by yellow dashed lines (in A’,B´,C´,D´,E). Discs are stained with (A-A´) anti-CycB (magenta), (B-B´) anti-PH3 (magenta), (C-C´) anti-BrdU (magenta), (D-D´) anti-CycE (magenta) and (E) *dap mRNA* (blue). The positions of the FMW and SMW are indicated by white and yellow arrowheads, respectively. White arrows in B’ and C’ point to the delayed re-entrance in cell cycle posterior to the SMW. Solid lines in D’ delimit the high levels of CycE within the MF in wild type cells. Scale bars represent 50μm.
(TIF)

S3 Fig *dac* mutant clones that fail to initiate a MF and PR differentiation show reduced Ptc levels, while those that initiate a MF and PR differentiation accumulates Ptc. All panels show standard confocal sections of L3 eye imaginal discs, containing *dac*^*3*^ mutant clones marked by the absence of GFP (green in A,B) and outlined by dashed line in A´, A´´,B´. Discs are stained with anti-Ptc (red) and anti-Elav (blue). Plain lines in A´ and B´ indicate the MF position. Scale bars represent 50μm.
(TIF)

S4 Fig Dac ChIP-seq. (A) Example gene (*nej*) with a Dac ChIP peak, 3kb upstream of the transcription start site. (B) Sequence logo of a candidate Dac motif found as the most enriched motif in the distal ChIP-peak. (C) Sequence logo of a Gli consensus binding site (annotated in the transfac database: transfac_pro-M01037) found significantly enriched in the set of distal ChIP-peaks when compared to random background sequences (p<0.001). (D) List of the proposed Dac targets, predicted by the presence of a nearby (<5kb from TSS or intronic) ChIP-seq peak, and the presence of the candidate Dac motif, as predicted by i-cisTarget [92].
(TIF)

S5 Fig *dac* is not required for *hh* or *ci* expression. (A-C´) Standard confocal sections of L3 eye imaginal discs (B-B´) wild type or (A-A´´´,C-C´) containing *dac*^*3*^ clones labeled with GFP (green in A,C) and outlined by dashed lines in A´-A´´´,C´, stained with (A-A´´´) anti-Elav (blue in A,A´, white in A´´´) and anti—β-Galactosidase to reveal *dppZ* (red in A-A´ and white in A´´) or (B-B´) anti-β-Galactosidase to reveal *ciZ* (green) and DAPI (magenta in B) or (C-C´) anti-β-Galactosidase to reveal *ciZ* (red in C and white in C´) and anti-ELAV (blue in C). (D-F,H-J) Standard confocal sections of L3 wing imaginal discs with anterior to the left and dorsal up in which *ptc*-Gal4 drives the expression of UAS-*mCherry* (green in D,E,F,H,I,J) and (H,I-J) UAS-*HA*::*dac*. Discs are stained with anti-Dac (red in D,H) and anti-β-Galactosidase to reveal *ciZ* (blue in D,H and magenta in E-F,I-J). E-E´ and I-I´ are magnifications of the dashed white square in D and H, respectively. F and J correspond to the area delimited by white dashed lines in E and I, respectively. The dashed yellow lines in E´ and I´ outline the AP compartment boundaries. (G,K) Profiles of the mCherry (green lines) and *ciZ* (magenta lines) intensity signals across the AP axis in F and J, respectively. Scale bars represent 50μm.
(TIF)
