# Supplementary figures and images for: dachshund Potentiates Hedgehog Signaling during Drosophila Retinogenesis
Source: PLoS Genet. 2016 Jul 21;12(7):e1006204. doi: 10.1371/journal.pgen.1006204 (PMC4956209; doi:10.1371/journal.pgen.1006204)

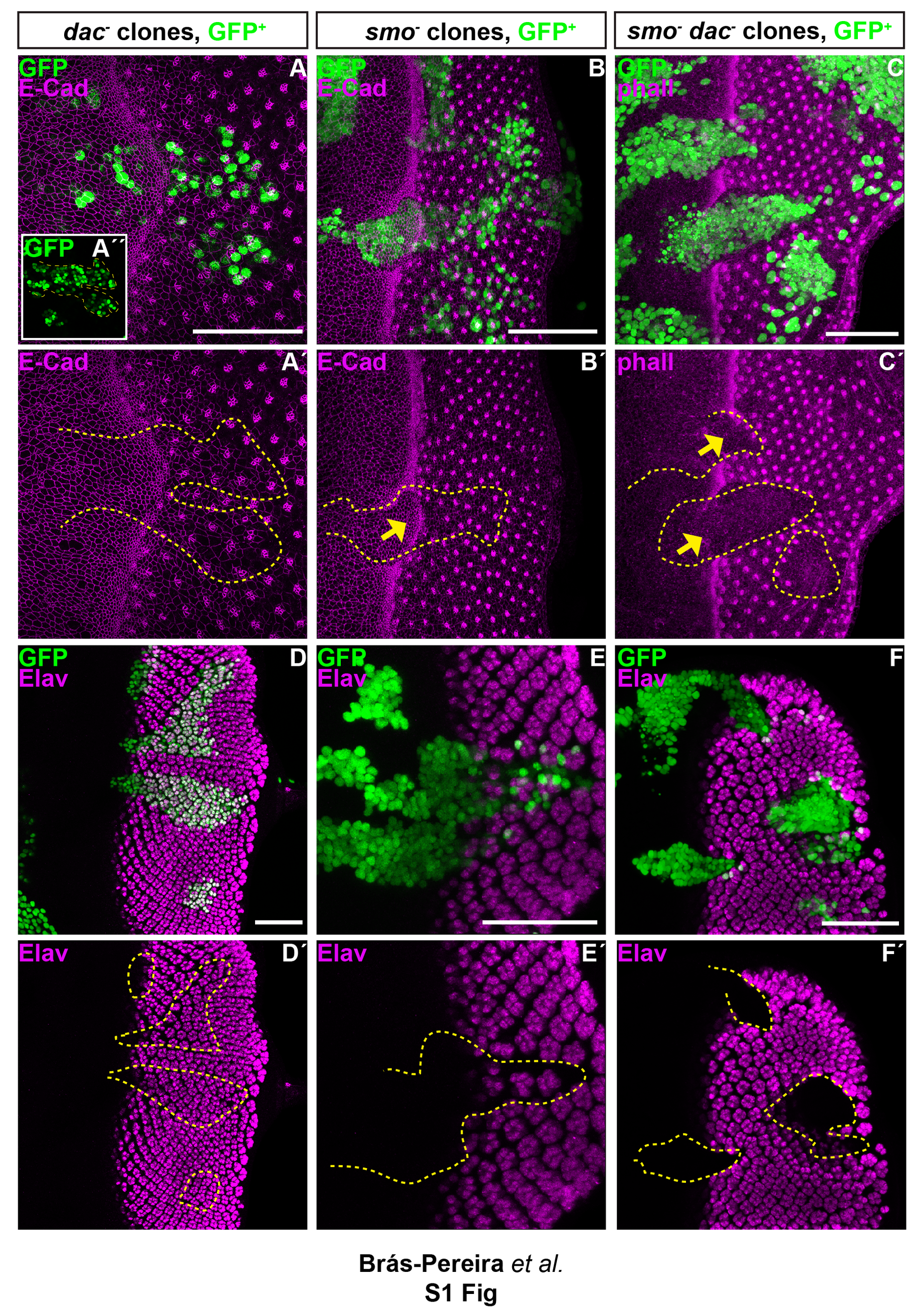

Supplement: S1 Fig — All panels show standard confocal sections of L3 eye imaginal discs, containing (A-A´´,D-D´) dac3 or (B-B´,E-E´) smo3 single mutant clones or (C-C´,F-F´) smo3, dac3 double mutant clones marked by the presence of GFP (green in A, A´´,B,C,D,E,F) and outlined by dashed line (in A´, A´´,B´,C´,D´,E´,F´). Discs are stained with (A-B´) anti-E-Cad (magenta) or (C-C´) phalloidin (magenta) to outline cell shape or with (D-F’) anti-Elav (magenta) to label differentiating PRs. A´´ corresponds to a merge between the GFP signal in A and more basal sections. Note that while loss of smo function has little effect on MF formation (arrow in B´) and PR differentiation, loss of both smo and dac function completely disrupts MF formation (arrows in C´) and PR differentiation. Scale bars represent 50μm. (TIF) [file pgen.1006204.s001.tif]

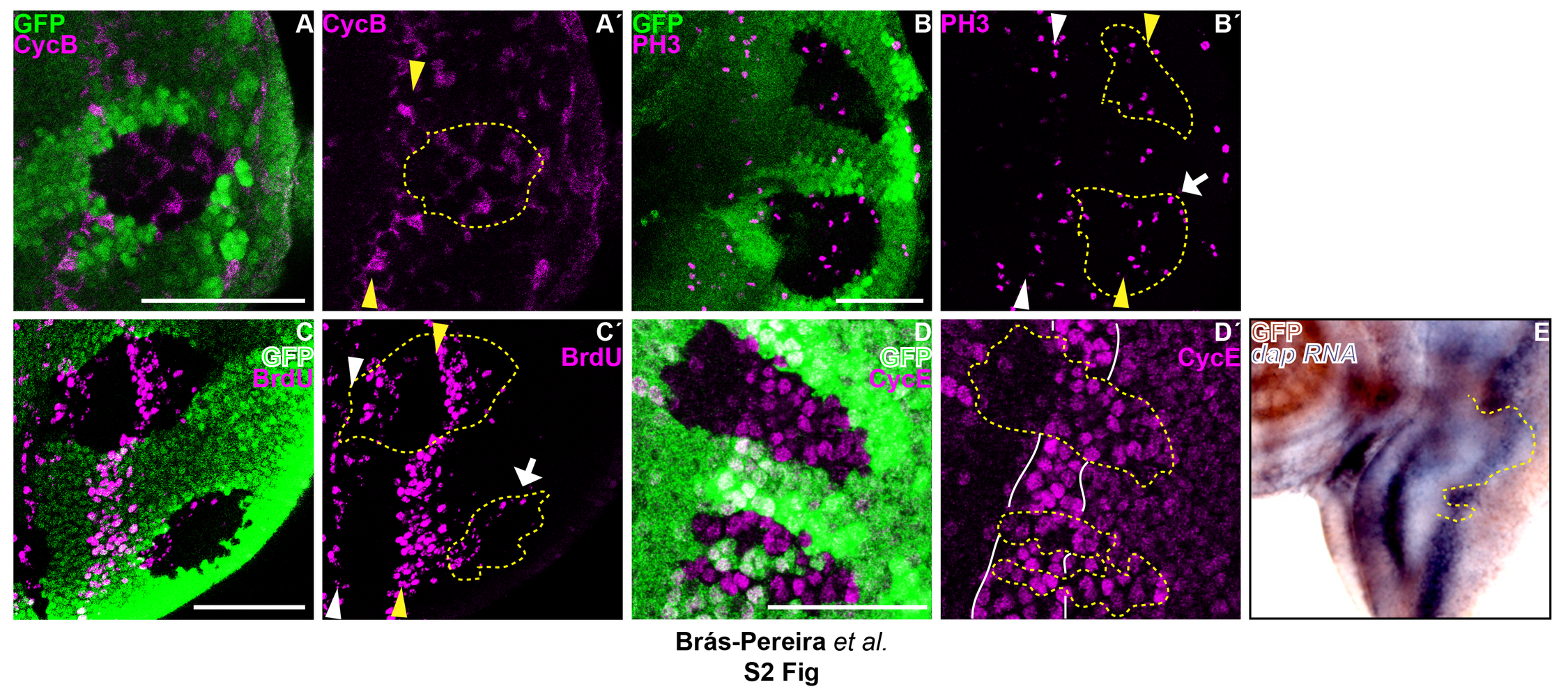

Supplement: S2 Fig — All panels show (A-D´) standard confocal sections or (E) standard brightfield image of L3 eye discs, containing dac3 mutant clones marked by the absence of GFP (green in A,B,C,D and brown in E) and outlined by yellow dashed lines (in A’,B´,C´,D´,E). Discs are stained with (A-A´) anti-CycB (magenta), (B-B´) anti-PH3 (magenta), (C-C´) anti-BrdU (magenta), (D-D´) anti-CycE (magenta) and (E) dap mRNA (blue). The positions of the FMW and SMW are indicated by white and yellow arrowheads, respectively. White arrows in B’ and C’ point to the delayed re-entrance in cell cycle posterior to the SMW. Solid lines in D’ delimit the high levels of CycE within the MF in wild type cells. Scale bars represent 50μm. (TIF) [file pgen.1006204.s002.tif]

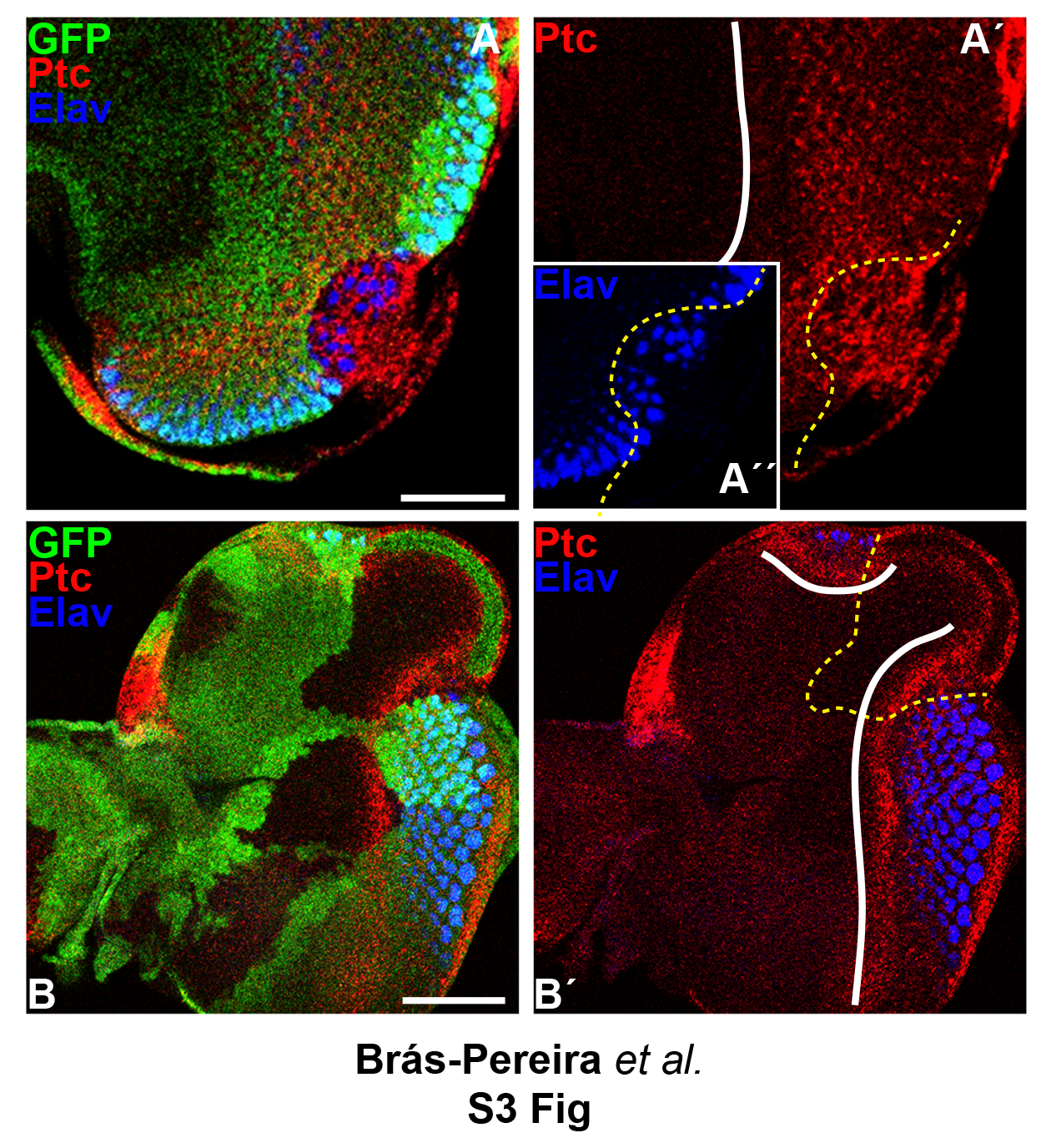

Supplement: S3 Fig — All panels show standard confocal sections of L3 eye imaginal discs, containing dac3 mutant clones marked by the absence of GFP (green in A,B) and outlined by dashed line in A´, A´´,B´. Discs are stained with anti-Ptc (red) and anti-Elav (blue). Plain lines in A´ and B´ indicate the MF position. Scale bars represent 50μm. (TIF) [file pgen.1006204.s003.tif]

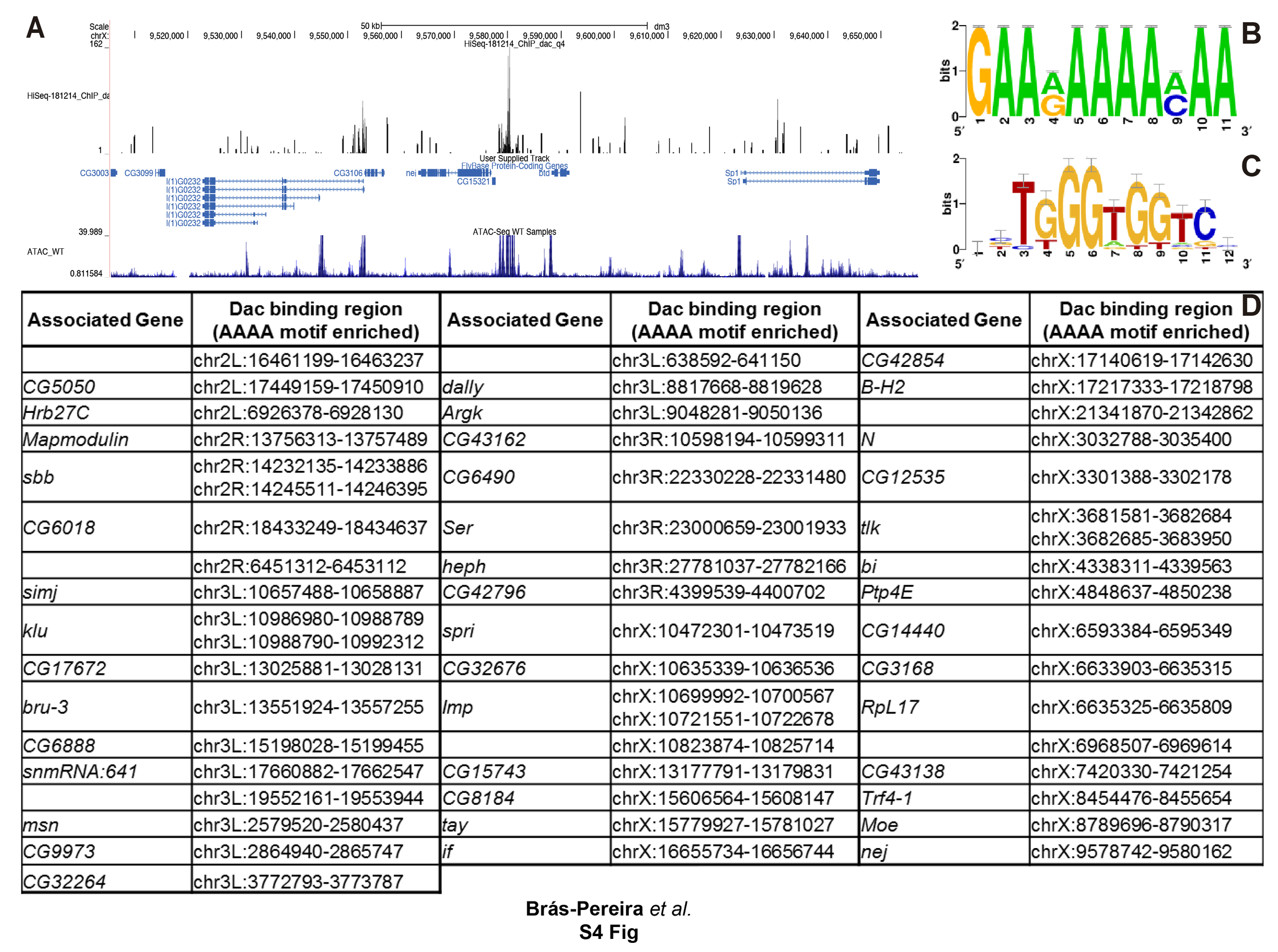

Supplement: S4 Fig — (A) Example gene (nej) with a Dac ChIP peak, 3kb upstream of the transcription start site. (B) Sequence logo of a candidate Dac motif found as the most enriched motif in the distal ChIP-peak. (C) Sequence logo of a Gli consensus binding site (annotated in the transfac database: transfac_pro-M01037) found significantly enriched in the set of distal ChIP-peaks when compared to random background sequences (p<0.001). (D) List of the proposed Dac targets, predicted by the presence of a nearby (<5kb from TSS or intronic) ChIP-seq peak, and the presence of the candidate Dac motif, as predicted by i-cisTarget [92]. (TIF) [file pgen.1006204.s004.tif]

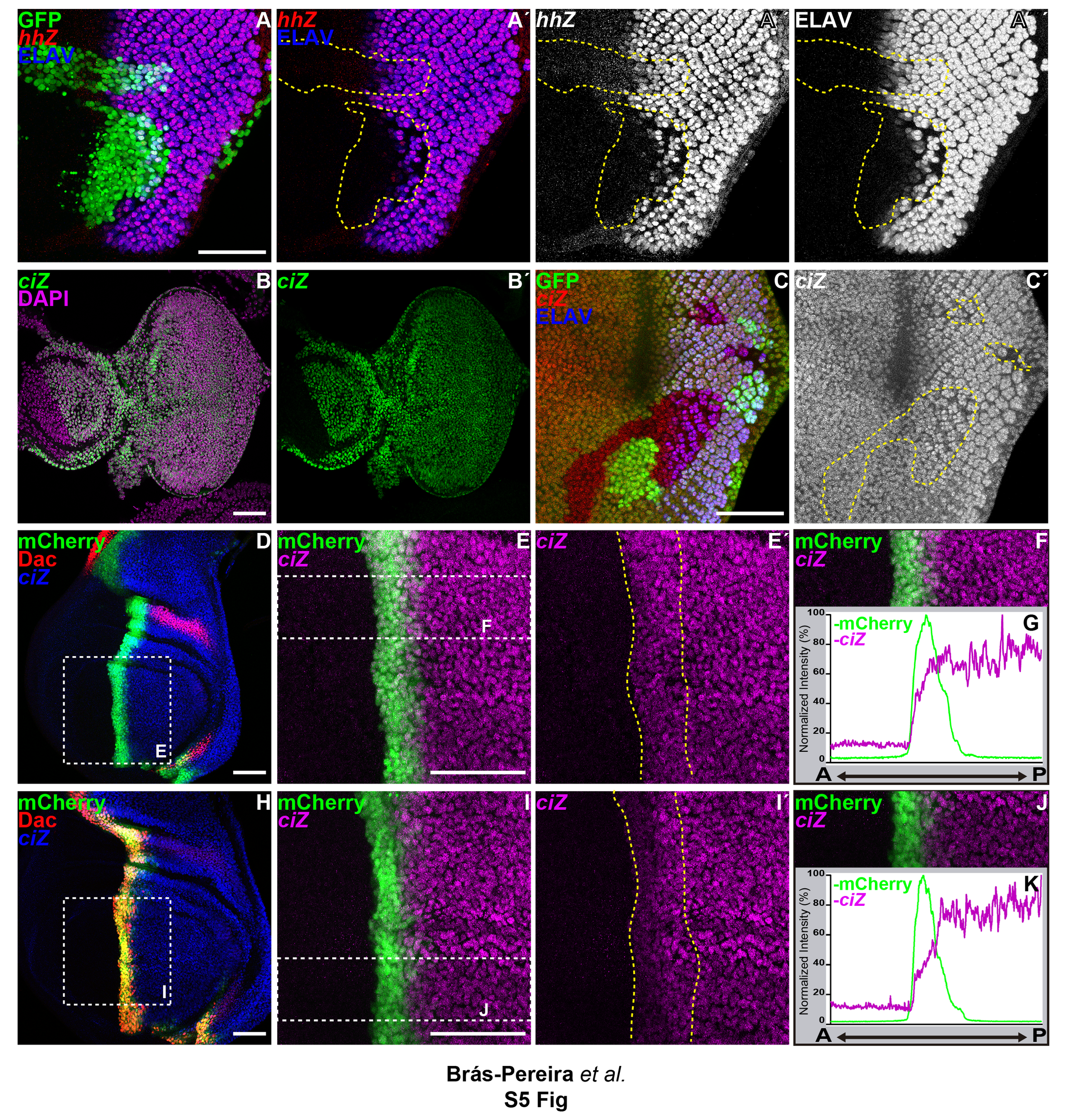

Supplement: S5 Fig — (A-C´) Standard confocal sections of L3 eye imaginal discs (B-B´) wild type or (A-A´´´,C-C´) containing dac3 clones labeled with GFP (green in A,C) and outlined by dashed lines in A´-A´´´,C´, stained with (A-A´´´) anti-Elav (blue in A,A´, white in A´´´) and anti—β-Galactosidase to reveal dppZ (red in A-A´ and white in A´´) or (B-B´) anti-β-Galactosidase to reveal ciZ (green) and DAPI (magenta in B) or (C-C´) anti-β-Galactosidase to reveal ciZ (red in C and white in C´) and anti-ELAV (blue in C). (D-F,H-J) Standard confocal sections of L3 wing imaginal discs with anterior to the left and dorsal up in which ptc-Gal4 drives the expression of UAS-mCherry (green in D,E,F,H,I,J) and (H,I-J) UAS-HA::dac. Discs are stained with anti-Dac (red in D,H) and anti-β-Galactosidase to reveal ciZ (blue in D,H and magenta in E-F,I-J). E-E´ and I-I´ are magnifications of the dashed white square in D and H, respectively. F and J correspond to the area delimited by white dashed lines in E and I, respectively. The dashed yellow lines in E´ and I´ outline the AP compartment boundaries. (G,K) Profiles of the mCherry (green lines) and ciZ (magenta lines) intensity signals across the AP axis in F and J, respectively. Scale bars represent 50μm. (TIF) [file pgen.1006204.s005.tif]
